# Supplementary material for: GenomeBits insight into omicron and delta variants of coronavirus pathogen
Source: PLoS One. 2022 Jul 11;17(7):e0271039. doi: 10.1371/journal.pone.0271039 (PMC9273097; doi:10.1371/journal.pone.0271039)
Supplement: Attachment — Submitted filename: Response_to_Reviewers.pdf [file pone.0271039.s002.pdf]

Response to Reviewers of PONE-D-22-01925

"GenomeBits insight into omicron and delta variants of coronavirus pathogen",
by E. Canessa, L. Tenze

We are indebted to the academic editor and the two reviewers for providing insightful comments on the manuscript. Our responds to each point raised are as follows:

- **Reply to Additional Editor Comments:**

The Introduction section was revised and completely rewritten. In particular, we added a discussion in the Introduction section about the origins of the SARS-Cov-2 pandemic as sugested by the Editor, and cited the three works suggested: *(i)* Boni, Maciej F., et al. *(ii)* Makarenkov, V., Mazoure, B., Rabusseau, G. et al., and *(iii)* Domingo JL. We also briefly mentioned the impact of the SARS-Cov-2 pandemic and added 11 new references to support our findings throughout the text. The abstract and conclusions have also been improved based on the data presented. All sequence data analysed here are publicly available at GSIAD and all custom code used in the manuscript is fully available at https://github.com/canessae/GenomeBits/ We have made an effort to present our manuscript in an intelligible fashion with an standard English. At revision, we corrected few typographical and grammatical errors.

- **Reply to Reviewer #1:**

1. We have made an effort to rewrite our manuscript and present it in an intelligible fashion with an standard English. Our statements now carry out associated new references and few sentences have been completed with a clear meaning.

2. We have added a new Table to aIllustrate the computation of binary scores for a given genomic sequence. This Table helps us to explain that in our case, the mapping into four binary projections of genome sequences carries alternating plus and minus signs, where + and - signs are chosen sequentially starting with +1 at k = 1 as default. The Table shows a particular mapping

example for converting the brief fragment AGATCTGTTCTC of 12 nucleotides into the alternating binary array. As a principal difference with previous binary representations (see, e.g., new Refs [19, 20]), our mapping (whose terms change sign –i.e., if a term X_k is positive then X_k+1 is negative and vice versa) displays their occurrence as +1 or -1 as well as their non existence as 0 at a given base pair k.

3. The inclusion of different smoothing sliding window sizes of up to about 500 bp (moving along the target genome sequences and repeating the GenomeBits procedure as described, lead to a data noise reduction in the curves and preserve the average behavior of the sums displayed in Figures 1 and 2.

4. Our results have been further elaborated. We emphasize that the regions of main discrepancies as found in the Similarity identities curves of Fig 1, e.g., around N=10000 are also reflected by the red lines of Fig 2 via GenomeBits. The main difference between both comparative genomics approaches is that changes via Eq (1) can be analyzed and characterized at each single A,C,G,T nucleotide level separately. Beside such comparision of Similarity studies and our previous analysis on Fast Fourier Transforms for coronavirus genome of other variants as reported in Ref[6], our present findings are new and have not similars to those reported by others. We report a kind of 'ordered' (constant) to 'disordered' (peaked) phase transition phenomena around the NSP5 polymerase within the open reading frames ORF1a region, up to the nucleotide region of the S-Protein. As seen in Fig 2, the black arrows indicate an analogous phase transition point appearing close to the coding region of the S-spike genes.

- **Reply to Reviewer #2:**

We particularly thanks this referee for finding our research article interesting and fully in the scope of PLOS One journal.

1. As suggested, the new Abstract now reads: “We apply the new GenomeBits method to uncover underlying genomic features of omicron and delta

coronavirus variants. This is a statistical algorithm whose salient feature is to map the nucleotide bases into a finite alternating (+-) sum series of distributed terms of binary (0,1) indicators. We show how by this method, distinctive signals can be uncovered out of the intrinsic data organization of amino acid progressions along their base positions. Results show a sort of *'ordered'* (or constant) to *'disordered'* (or peaked) transition around the coronavirus S-spike protein region. Together with our previous results for past variants of coronavirus: Alpha, Beta, Gamma, Epsilon and Eta, we conclude that the mapping into GenomeBits strands of omicron and delta variants can help to characterize mutant pathogens."

2. We have made an effort to present our manuscript in an intelligible fashion with an standard English. At revision, we corrected few typographical and grammatical errors.

3. (idem as 2)

4. As also replied to the Academic Editor, the Introduction section was revised and completely rewritten. In particular, we added a discussion in the Introduction section about the origins of the SARS-Cov-2 pandemic and cited several new references. The abstract and conclusions have also been improved based on the data presented

5. In the Conclusion section we now provide a general reinterpretation of the results in the context of the present study and other evidence published, and also briefly discussed implications for future research.

-oOo-
